# Efficacy of two endectoparasiticide products combining fipronil and (S)-methoprene or esafoxolaner with eprinomectin and praziquantel against fleas and intestinal helminths in cats naturally infested in Brazil

**DOI:** 10.1051/parasite/2022012

**Published:** 2022-03-03

**Authors:** Andre A. Cutolo, Debora T.G. Jardina, Gabriela G. De Vito, Cristiano Grisi do Nascimento, Maycon Junior Heidmann, José Dirceu Ferreira Nantes, Karolyne Vieira Bassetto, Isabella Cristina Chagas, Edgar Ferreira Pereira Junior, Bruno Gomes de Castro, Eric Tielemans

**Affiliations:** 1 Boehringer-Ingelheim Animal Health, Fazenda São Francisco 13140-000 Paulinia SP Brazil; 2 ICON Global Strategic Solutions 1308 avenue Doutor Cardoso de Melo 04548-004 São Paulo SP Brazil; 3 Convolution Divisão de Antiparasitários 150 Lauro Valente Street, Cond Saint Gerard 14022-048 Ribeirão Preto SP Brazil; 4 Universidade Federal de Mato Grosso 1200 Alexandre Ferronato Avenue 78557-267 Sinop MT Brazil; 5 Boehringer-Ingelheim Animal Health 29 avenue Tony Garnier 69007 Lyon France

**Keywords:** Cat, Efficacy, Flea, Frontline^®^ Protect/Broadline^®^, Intestinal helminth, NexGard^®^ Combo

## Abstract

Eprinomectin and praziquantel, nematodicide and cestodicide compounds, are both combined with the insecticide and acaricide compounds fipronil and (S)-methoprene in Frontline^®^ Protect/Broadline^®^, or esafoxolaner in NexGard^®^ Combo. These topical feline endectoparasiticide products were tested for efficacy against fleas and intestinal helminths in a field trial in Brazil. Flea- and/or helminth-infested domestic cats were treated twice at a monthly interval following label instructions: 160 cats with Frontline^®^ Protect/Broadline^®^ and 165 cats with NexGard^®^ Combo. The flea and intestinal helminth infestations were evaluated using comb counts and copromicroscopy, respectively before first treatment for baseline value, then 9 and 30 days after each treatment for fleas, and 9 days after each treatment for helminths. Multiparasitism was very frequent at baseline, as amongst the 325 included cats, 295, 280, 86 and 93 cats were at least infested with *Ctenocephalides* fleas, *Ancylostoma, Toxocara* and *Dipylidium caninum*, respectively. Efficacies were calculated by comparing the geometric means at baseline and at post-treatment timepoints for each parasite genus/species. Inclusive of both products and of all evaluation timepoints, the *Ctenocephalides*, *Ancylostoma, Toxocara* and *D. caninum* efficacies were at least 98.3%, 99.8%, 99.8% and 96.3%, respectively. No adverse reactions were observed, except for a few instances of mild, transient, and self-resolving hypersalivation occurring on the day of treatment in both groups. This field trial demonstrated high-level efficacy of Frontline^®^ Protect/Broadline^®^ and NexGard^®^ Combo against major parasites of cats in Brazil.

## Abstract (fr)

L’éprinomectine et le praziquantel, composés nématodicides et cestodicides, sont tous les deux associés aux composés insecticides et acaricides fipronil et (S)-méthoprène dans Frontline^®^ Protect/Broadline^®^, ou esafoxolaner dans NexGard^®^ Combo. Ces produits endectoparasiticides félins topiques ont été testés pour leur efficacité contre les puces et les helminthes intestinaux lors d’un essai sur le terrain au Brésil. Des chats domestiques infestés de puces et/ou d’helminthes ont été traités deux fois à intervalle d’un mois en suivant les instructions d’utilisation, 160 chats avec Frontline^®^ Protect/Broadline^®^ et 165 chats avec NexGard^®^ Combo. Les infestations par les puces et les helminthes intestinaux ont été évaluées en utilisant respectivement par comptage au peigne et par copromicroscopie, avant le premier traitement pour la valeur de base, puis 9 et 30 jours après chaque traitement pour les puces, et 9 jours après chaque traitement pour les helminthes. Le multiparasitisme était très fréquent à l’inclusion puisque parmi les 325 chats inclus, 295, 280, 86 et 93 chats étaient au moins infestés respectivement par les puces *Ctenocephalides*, ou *Ancylostoma*, *Toxocara* et *Dipylidium caninum*. Les efficacités ont été calculées en comparant les moyennes géométriques au départ et aux points d’évaluation post-traitement pour chaque genre/espèce de parasite. En incluant les deux produits et tous les points temporels d’évaluation, les efficacités contre *Ctenocephalides*, *Ancylostoma*, *Toxocara* et *D. caninum* étaient respectivement d’au moins 98,3 %, 99,8 %, 99,8 % et 96,3 %. Aucun effet indésirable n’a été observé à l’exception de quelques cas d’hypersalivation légère, transitoire et auto-résolvante survenant le jour d’un traitement dans les deux groupes. Cet essai sur le terrain a démontré une efficacité de haut niveau de Frontline^®^ Protect / Broadline^®^ et NexGard^®^ Combo contre les principaux parasites des chats au Brésil.

## Introduction

Frontline^®^ Protect/Broadline^®^ (abbreviated as “Broadline^®^” in the rest of the document), a combination of fipronil, (S)-methoprene, eprinomectin and praziquantel; and NexGard^®^ Combo, a combination of esafoxolaner, eprinomectin and praziquantel are both topical endectoparasiticide products for cats aimed at the treatment and control of multiparasitism, a common condition in the feline species [2, 4, 16, 21]. Fipronil/(S)-methoprene and esafoxolaner provide insecticide and acaricide properties, eprinomectin and praziquantel provide nematodicide and cestodicide properties, respectively. Broadline^®^ has been used safely for several years [8] and was demonstrated to be efficacious against a broad spectrum of feline parasites, including fleas [1] and gastro-intestinal helminths [13, 24]. NexGard^®^ Combo was recently developed and registered in the European Union [9] and was demonstrated to be efficacious against fleas in experimental infestation studies conducted with isolates originating from France, South Africa and the USA [25], and in field studies with naturally infested cats from Europe, the USA and Australia [27], and against gastro-intestinal helminths using induced and natural models of infection with isolates originating from Europe, North America and South Africa [12]. The safety of NexGard^®^ Combo was also comprehensively demonstrated, namely in specific target animal safety studies [10, 26].

It was necessary to confirm the efficacy of these two products against major feline parasites in South America, as the control of feline parasitism is important worldwide both for veterinary and public health [6, 28], and as authorities in major regions of the world require efficacy testing on local strains of parasites for registration of new products. This manuscript describes a field trial conducted in the Brazilian Southern Amazonian region for the investigation of the efficacy and tolerance of Broadline^®^ and NexGard^®^ Combo in cats naturally infested with fleas and/or intestinal helminths.

## Materials and methods

### Ethics

The study protocol had been reviewed and approved by the sponsor’s institutional animal care and use committee, and a license had been obtained from the local authorities.

### Study design

This study was designed in accordance with the “World Association for the Advancement of Veterinary Parasitology (W.A.A.V.P.) guidelines for evaluating the efficacy of parasiticides for the treatment, prevention and control of flea and tick infestation on dogs and cats” [17], the “WAAVP guidelines for evaluating the efficacy of anthelmintics for dogs and cats” [11], the International Cooperation on Harmonisation of Technical Requirements for Registration of Veterinary Medicinal Products VICH GL7, “Efficacy of Anthelmintics: General Requirements” [31], and VICH GL20 “Efficacy of Anthelmintics: Specific Recommendations for Felines” [32]. The study was conducted in accordance with Good Clinical Practices as described in “International Cooperation on Harmonization of Technical Requirements for Registration of Veterinary Medicinal Products (VICH) guideline GL9”.

The objectives of the study were to confirm the safety and efficacy of Broadline^®^ and NexGard^®^ Combo after one and two successive monthly treatments, against fleas and intestinal helminths naturally acquired in the field in Brazil.

The study was conducted on domestic cats in urban areas of Mato Grosso and Pará states, Brazil. In Mato Grosso, 83 and 85 cats, and in Pará, 77 and 79 cats, were assigned to the Broadline^®^ and NexGard^®^ Combo groups, respectively.

To qualify for the study, a household had at least one cat naturally infested with six or more live fleas and/or intestinal helminth(s) (*Ancylostoma* and/or *Toxocara* eggs, and/or *Dipylidium* eggs or proglottids). A household could contain a maximum of five dogs and cats (to minimize environmental flea infestation bias, dogs were administered oral afoxolaner), and all cats from a household were treated with the same product. The cat or cats adequately infested were evaluated for efficacy and safety; any other cat with an insufficient or absent infestation was only evaluated for safety. Neither the cat(s) nor the environment had been treated with any parasiticide compound within two months of inclusion.

Allocation into the Broadline^®^ or NexGard^®^ Combo groups was performed by lottery (taking numbers out of a hat), being performed on blocks of two households. If the first household was randomized into one treatment, consequently the second was allocated to the other treatment group. The process was repeated for each new pair of households containing included cats. A total of 160 cats were included in the Broadline^®^ group and 165 cats in the NexGard^®^ Combo group. There was only one cat in a multi-pet household assigned to NexGard^®^ Combo that did not meet the infestation criteria; this cat was therefore treated, but evaluated only for safety.

Ninety percent of these 325 cats were infested with more than one parasite. In the Broadline^®^ group, 146, 143, 42 and 46, and in the NexGard^®^ Combo group, 149, 137, 44 and 47 evaluations of flea, *Ancylostoma, Toxocara* and *Dipylidium* infestations were conducted, respectively (Table 3).

#### Animals

To qualify for inclusion, cats were to harbor a minimum of six fleas and/or helminth eggs/proglottids in feces, and were declared suitable for the study in terms of health by a veterinarian. The 325 included domestic cats were aged 2 months to 13 years, weighed 0.6–6.1 kg, and were not limited by sex and reproductive status (neutered or intact). They were predominantly Domestic Short/or Long Hair, except for 9 Siamese, 3 Turkish Angora, and 1 Persian. Cats lived indoors, outdoors, or had both indoor and outdoor access; no housing restrictions were implemented and cats were fed as usual.

#### Treatment

Cats were treated by the Investigator on Days 0 and 30 (±2) at the recommended label dose of 0.3 mL (for cats weighing 0.8 to <2.5 kg), or 0.9 mL (for cats weighing 2.5 to <7.5 kg), which for Broadline^®^ corresponded to 10.0–31.1 mg/kg fipronil, 12–37.5 mg/kg (S)-methoprene, 0.48–1.50 mg/kg eprinomectin, and 10.0–31.1 mg/kg praziquantel, and for NexGard^®^ Combo 1.44–4.5 mg/kg esafoxolaner, 0.48–1.50 mg/kg eprinomectin, and 10.0–31.1 mg/kg praziquantel. The treatments were applied as per respective labels, in one spot directly on the skin, after parting the hair, in the midline of the neck between the base of the skull and the shoulder blades.

#### Parasite counts

Flea counts were performed by systematically combing all parts of the cat using a fine-tooth flea comb for at least 5 min. The counts were performed before the first treatment for baseline evaluation, then on Days 9 (±2), 30 (before second treatment), 39 and 60 for efficacy evaluations.

Intestinal helminth evaluations (*Ancylostoma sp*., *Toxocara sp*. and *Dipylidium sp.*) were performed by copromicroscopy using McMaster and Centrifugal Flotation Techniques, for the expression of egg counts in eggs per gram (EPG). *Dipylidium sp.* evaluations were also performed by visual examination and count of proglottids in feces. The evaluations were performed on fecal samples obtained before the first treatment for baseline evaluation and inclusion, then on Days 9 (±2) and 39. Fecal samples were collected at the households, with proper care taken to confirm the origin in case of multi-cat households.

#### Tolerance evaluations

Owners were requested to observe their cats closely for 2 h after each treatment, then daily, and to report any abnormality to the veterinarian in charge. At each scheduled visit (Days 0, 9, 30, 39 and (flea only) 60), the Investigator performed a physical examination, which together with consideration of any adverse reactions reported by owners (resulting or not in an unplanned veterinary consultation, veterinary care, concurrent medication, etc.) was considered for an evaluation of the tolerance. Relationships to treatment for all adverse reactions and abnormalities were evaluated by the Investigator.

### Statistical analysis

Total live flea counts, EPG and proglottid counts were transformed to the natural logarithm of (count + 1) for analysis and calculation of the geometric means. The percent efficacy was calculated using the formula 100 × [(*B* – *T*)/*B*], where *B* = geometric mean of the Day 0 (baseline) visit count and *T* = geometric mean of the appropriate visit day count. The geometric mean was calculated by taking the anti-logarithm of the average of the log-counts and then subtracting 1, or was computed by taking the anti-logarithm of the least square mean minus 1 from the analysis model. Flea efficacy was also calculated on the basis of arithmetic means.

## Results

### Flea and intestinal helminth efficacies

The flea efficacy results are presented in Table 1.

**Table 1:** Flea efficacy results.

		Day 0	Day 9	Day 30	Day 39	Day 60
	*n* cats	146	146	146	146	146
Broadline^®^	Geo Mean	11.4	0.2	0.2	0.0	0.1
	% efficacy		98.5	98.3	99.7	99.4
	*p*-value		<0.0001	<0.0001	<0.0001	<0.0001
	A Mean	12.2	0.3	0.4	0.1	0.1
	% efficacy		97.5	96.4	99.5	99.2
	*n* cats	149	149	149	149	149
NexGard^®^ Combo	Geo Mean	10.9	0.11	0.09	0.01	0.02
	% efficacy		99.0	99.2	99.9	99.8
	*p*-value		<0.0001	<0.0001	<0.0001	<0.0001
	A Mean	11.5	0.19	0.15	0.02	0.03
	% efficacy		98.4	98.7	99.8	99.7

Geo Mean = geometric mean of flea counts; A Mean = arithmetic mean of flea counts.

1 Percent efficacy = 100[(*C* − *T*)/*C*], where *C* is the geometric (Geo) mean at baseline (Day 0) and *T* is the geometric mean at evaluation timepoints.

2 Percent efficacy = 100[(*C* − *T*)/*C*], where *C* is the arithmetic (*A*) mean at baseline (Day 0) and *T* is the geometric mean at evaluation timepoints.

3 Two-sided probability value from analysis of variance on log-counts of the mean at baseline and evaluation timepoints.

In the Broadline^®^ group, the 146 cats evaluated for fleas were infested with 6–43 fleas at inclusion (geometric mean 11.4), and the percent reduction of live fleas compared to baseline, 9 and 30 days after each of the two treatments ranged from 98.3% to 99.7%.

In the NexGard^®^ Combo group, the 149 cats evaluated for fleas were infested with 6–31 fleas at inclusion (geometric mean 10.9), and the percent reduction of live fleas compared to baseline, 9 and 30 days after each of the two treatments ranged from 99.0% to 99.9%.

The intestinal helminths efficacy results are presented in Table 2.

**Table 2:** Gastro-intestinal helminths efficacy results.

		*Ancylostoma*			*Toxocara*			*Dipylidium caninum*		
		Day 0	Day 9	Day 39	Day 0	Day 9	Day 39	Day 0	Day 9	Day 39
Broadline^®^	*n* cats	143	143	143	42	42	42	46	46	46
	Geo Mean	459.2	0.9	0.3	190.9	0.2	0.1	1.6	0.0	0.0
	% efficacy		99.8	99.9		99.9	99.9		98.0	97.0
	*p*-value		<0.0001	<0.0001		<0.0001	<0.0001		<0.0007	<0.0012
NexGard^®^ Combo	*n* cats	137	137	137	44	44	44	47	47	47
	Geo Mean	426.2	0.6	0.3	202.8	0.4	0.2	1.65	0.06	0.03
	% efficacy		99.9	99.9		99.8	99.9		96.3	98.2
	*p*-value		<0.0001	<0.0001		<0.0001	<0.0001		<0.0009	<0.0006

Geo Mean = geometric mean of eggs per gram (EPG) for *Ancylostoma* and *Toxocara*, and proglottids for *Dipylidium*.

1 Percent efficacy = 100[(*C* − *T*)/*C*], where *C* is the geometric (Geo) mean at baseline (Day 0) and *T* is the geometric mean at evaluation timepoints.

2 Two-sided probability value from analysis of variance on log-counts of the mean at baseline and evaluation timepoints.

In the Broadline^®^ group, the helminth efficacies evaluated nine days after each treatment exceeded 99% for nematodes (*Ancylostoma* and *Toxocara*) and 97% for *Dipylidium*.

In the Nexgard^®^ Combo group, the helminth efficacies evaluated nine days after each treatment exceeded 99% for nematodes (*Ancylostoma* and *Toxocara*) and 96% for *Dipylidium*.

At inclusion, inclusive of both groups, most cats were infested by fleas (91.0%), by helminths (94.8%), and were mixed infested by fleas and helminths (85.8%). The types and proportions of infestations including the mixed infestations are summarized in Table 3 and detailed in Table 4.

**Table 3:** Parasitism of cats and types of mixed infestations.

	Broadline^®^		NexGard Combo^®^		Total	
	*n*	%	*n*	%	*n*	%
Fleas	146	91.3	149	90.9	295	91.0
Helminths	154	96.3	153	93.3	307	94.8
Nematodes	146	91.3	141	86.0	287	88.6
Cestodes	46	28.8	47	28.7	93	28.7
At least two parasites	148	92.5	144	87.8	292	90.1
At least three parasites	65	40.6	68	41.5	133	41.0
Fleas and helminths	140	87.5	138	84.1	278	85.8

**Table 4:** Details of infestations at baseline.

	Broadline^®^		NexGard^®^ Combo		Total	
	*n*	%	*n*	%	*n*	%
Fleas only	6	3.8	11	6.7	17	5.2
*Ancylostoma* only	6	3.8	8	4.9	14	4.3
*Toxocara* only	0	0.0	0	0.0	0	0.0
*Dipylidium* only	0	0.0	1	0.6	1	0.3
*Ancylostoma* and *Toxocara*	7	4.4	4	2.4	11	3.4
*Ancylostoma* and *Dipylidium*	1	0.6	0	0.0	1	0.3
*Toxocara* and *Dipylidium*	0	0.0	2	1.2	2	0.6
*Ancylostoma* and *Toxocara* and *Dipylidium*	0	0	0	0	0	0
Fleas and *Ancylostoma*	64	40.0	58	35.4	122	37.7
Fleas and *Toxocara*	3	1.9	1	0.6	4	1.2
Fleas and *Ancylostoma* and *Toxocara*	28	17.5	35	21.3	63	19.4
Fleas and *Dipylidium*	8	5.0	11	6.7	19	5.9
Fleas and *Ancylostoma* and *Dipylidium*	33	20.6	31	18.9	64	19.8
Fleas and *Toxocara* and *Dipylidium*	0	0.0	1	0.6	1	0.3
Fleas and *Ancylostoma* and *Toxocara* and *Dipylidium*	4	2.5	1	0.6	5	1.5
Total number of cats	160		164		324	

*n* = number of cats diagnosed positive for the parasite at baseline (before first treatment).

#### Tolerance

A total of 320 Broadline^®^ and 330 NexGard^®^ Combo treatments were administered.

In the Broadline^®^ group, nine occurrences of mild and self-resolving hypersalivation were observed and lasted up to 40 min in eight instances, and 24 h one instance. No other adverse reactions were observed.

In the NexGard^®^ Combo group, five occurrences of mild and self-resolving hypersalivation were observed and lasted up to 40 min. No other adverse reactions were observed.

No animal needed or received any concurrent medication during the study.

## Discussion and conclusion

This field trial confirmed that Broadline^®^ or NexGard^®^ Combo applied to cats in Brazil have a comparable and high level of efficacy against fleas, *Ancylostoma* and *Toxocara* infestations. The results also showed high efficacy against *D. caninum* infections for both products, however, less conclusively as the detection methods used (copromicroscopy and visual examination of proglottids on feces) lack sensitivity and have been demonstrated to only reveal a fraction of the true infection incidence when compared to worm counts performed under necropsy [15]. Comparable efficacy was fully expected for both products with regards to intestinal helminths, as their active ingredients, eprinomectin and praziquantel, are identical and administered at the same dosage. Comparable efficacy was expected to a lesser extent with regards to fleas, as on the one hand esafoxolaner, the isoxazoline ectoparasiticide active ingredient of NexGard^®^ Combo, is a novel systemic compound to which no fleas have yet been exposed in Brazil, while on the other fipronil, the ectoparasiticide active ingredient of Broadline^®^, is a contact compound that has been used worldwide for several decades, including in Brazil, in several animal species, public hygiene, and also in crop agriculture. The high efficacy results of fipronil (in combination to (S)-methoprene) observed in the present field trial show that this ingredient remains a good tool to control fleas in Brazil.

This trial also confirmed that cats are a species commonly infested by multiple parasites, as referenced in Brazil [14, 18, 19, 23] and worldwide [2–4, 6, 7, 15, 21]. In the present study, 85.8% of domestic cats from an urban area were simultaneously infested with fleas and intestinal helminths, and 21.6% with fleas and intestinal nematodes and cestodes (Table 3). There was a gap in the epizootiological investigation of this study, as the incidences of *Aelurostrongylus abstrusus* and *Troglostrongylus brevior*, major lungworms of cats were not investigated. The specific diagnostic technique for *A. abstrusus* and *T. brevior* (Baermann funnel migration) was not performed due to study logistics; nevertheless, the worldwide importance [20, 29, 30] including in Brazil [5, 22] of these feline lungworms should not be overlooked by the epizootiological observations of the present study.

These observations confirm the importance of using a broad endectoparasiticide medical strategy in cats, including ectoparasiticide, nematodicide and cestodicide treatments.

This field trial demonstrated a high level of tolerance and efficacy of Broadline^®^ and NexGard^®^ Combo against major parasites of cats in Brazil.

## Competing interest

The work reported herein was funded by Boehringer-Ingelheim. The authors are current employees or contractors of Boehringer-Ingelheim. Other than that, the authors declare no conflict of interest. This document is provided for scientific purposes only. Any reference to a brand or trademark herein is for information purposes only and is not intended for any commercial purposes or to dilute the rights of the respective owners of the brand(s) or trademark(s).
